# ID 214 - Validação da Metodologia DEA (*Data Envelopment Analysis*) para Avaliação de Eficiência Operacional em Sistemas de Saúde

**DOI:** 10.5327/2237-9622.2025.v34s1.214

**Published:** 2025-11-25

**Authors:** Lívia Loamí Ruyz Jorge de Paula, Felipe Ribeiro Cabral Fagundes, Bruno Tirotti Saragiotto

**Keywords:** *Data Envelopment Analysis* (DEA), eficiência operacional, sistemas de saúde, custo-efetividade, gestão hospitalar

## Abstract

**Introdução:**

A sustentabilidade dos sistemas de saúde no Brasil tem sido um desafio crescente, especialmente em hospitais de alta complexidade e operadoras de saúde, devido aos altos custos operacionais e desperdícios de recursos. A busca por métodos mais eficientes para avaliar a relação entre custos e desfechos clínicos tem se intensificado. Nesse contexto, a metodologia DEA () surge como uma ferramenta inovadora para medir a eficiência dos sistemas de saúde, fornecendo subsídios para uma gestão mais eficaz dos recursos hospitalares.

**Método:**

A metodologia do estudo foi dividida em cinco fases principais: preparação e integração tecnológica: implementou-se a plataforma tecnológica Hi! em hospitais e operadoras de saúde, como o Hospital de Amor e a Unimed, para integrar os dados financeiros e clínicos; coleta e análise de dados: realizou-se a coleta contínua de dados sobre custos de tratamentos e desfechos clínicos para aplicação da metodologia DEA, e os dados foram comparados com os métodos tradicionais de análise de custo-efetividade; identificação de desperdícios: procedeu-se à análise dos processos hospitalares para identificar áreas de desperdício, como superutilização de recursos e ineficiências em procedimentos médicos; propostas de melhoria e implementação: foram sugeridas intervenções para otimizar os recursos identificados, promovendo maior eficiência operacional; avaliação final e disseminação dos resultados: avaliaram-se os impactos da metodologia nas instituições participantes, gerando relatórios detalhados e divulgando os resultados por meio de publicações científicas e apresentações em congressos. A coleta de dados foi realizada com base na plataforma digital desenvolvida pela Hi!, que permitiu a captura de informações detalhadas sobre custos e desfechos clínicos. A metodologia DEA foi aplicada para medir a eficiência dos hospitais e das operadoras na transformação de recursos financeiros em resultados clínicos positivos para os pacientes.

**Resultados:**

A validação da metodologia DEA resultou em uma redução significativa dos custos operacionais e promoveu uma gestão mais eficiente e sustentável dos recursos nos sistemas de saúde. O estudo estabeleceu um novo padrão para a avaliação de eficiência operacional em instituições de saúde no Brasil.

**Conclusão:**

A aplicação da DEA como metodologia prática e inovadora na análise de eficiência operacional transformou a gestão hospitalar no Brasil, melhorando a sustentabilidade financeira das instituições de saúde e otimizando a alocação de recursos.

